# Effect of Sweet Wormwood *Artemisia annua* Crude Leaf Extracts on Some Biological and Physiological Characteristics of the Lesser Mulberry Pyralid, *Glyphodes pyloalis*

**DOI:** 10.1673/031.011.15601

**Published:** 2011-11-14

**Authors:** Roya Khosravi, Jalal Jalali Sendi, Mohammad Ghadamyari, Elham Yezdani

**Affiliations:** Department of Plant Protection, College of Agriculture, University of Guilan, Rasht, Iran

**Keywords:** development, digestive enzymes, Glutathion S-transferase, physiology, toxicity

## Abstract

The lesser mulberry pyralid, *Glyphodes pyloalis* Walker (Lepidoptera: Pyralidae) is a monophagous and dangerous pest of mulberry that has been recently observed in Guilan province, northern Iran. In this study, the crude methanol extract of sweet wormwood *Artemisia annua* L. (Asterales: Asteracaea) was investigated on toxicity, biological and physiological characteristics of this pest under controlled conditions (24 ± 1 °C, 75 ± 5% RH, and 16:8 L:D photoperiod). The effect of acute toxicity and sublethal doses on physiological characteristics was performed by topical application. The LC_50_ and LC_20_ values on fourth instar larvae were calculated as 0.33 and 0.22 gram leaf equivalent/ mL, respectively. The larval duration of fifth instar larvae in LC_50_ treatment was prolonged (5.8 ± 0.52 days) compared with the control group (4.26 ± 0.29 days). However larval duration was reduced in the LC_20_ treatment. The female adult longevity in the LC_50_ dose was the least (4.53 ± 0.3 days), while longevity among controls was the highest (9.2 ± 0.29 days). The mean fecundity of adults after larval treatment with LC_50_ was recorded as 105.6 ± 16.84 eggs/female, while the control was 392.74 ± 22.52 eggs/female. The percent hatchability was reduced in all treatments compared with the control. The effect of extract in 0.107, 0.053, 0.026 and 0.013 gle/mL on biochemical characteristics of this pest was also studied. The activity of α-amylase and protease 48 hours post—treatment was significantly reduced compared with the control. Similarly lipase, esterase, and glutathione S-transferase activity were significantly affected by *A. annua* extract.

## Introduction

The lesser mulberry pyralid *Glyphodes pyloalis* Walker (Lepidoptera: Pyralidae) is a monophagous and dangerous pest of mulberry *Morus* sp. (Moraceae). This pest is a specialist insect on mulberry, and is widely distributed throughout Asia and the Northern Province of Iran, where the species cause serious damage to sericulture. The amount of food eaten by the first and second instar larvae is negligible, but feeding increases in later instars. Fourth and fifth instar larvae secrete fine threads to fold the leaf and feed on the mesophyll inside the folds, and fifth instar larvae feed on the whole leaf until only the ribs remain (Khosravi and Jalali Sendi 2010). The use of chemical pesticide is lessening due to the dangers of acute and chronic toxicity for sellers, farm workers, and consumers of agriculture products (Isman 2006). Botanical pesticides have been shown to have little impact on natural enemies (Lal and Mulla 1992; Smith and Stratton 1986; Schmutterer 1995, 1997), and therefore have the potential to be used in combination with biological control in the development of an integrated pest management system. The use of plant extracts in agroecosystems is now emerging as one of the prime means to protect crop products and the environment from synthetic pesticide pollution. In general, botanical pesticides have low mammalian toxicity, less impact on non-target organisms, and are easily available and less expensive than their synthetic counterparts (Prakash and Rao 1997; Schmutterer 1995).

The genus *Artemisia* belongs to the large family of Asteraceae, encompassing more than 300 species. *Artemisia annua* L. (Asterales: Asteracaea), commonly known as sweet wormwood or annual wormwood, grows widely in Europe and America and is planted to a large extent in China, Turkey, Vietnam, Afghanistan, and Australia (Bhakuni et al. 1988). These plants have antimalarial effects and are used as a clinical medicinal plant in many countries (Ridder et al. 2008). Some studies have reported insecticidal effects of *A. annua* extract including growth retardation, as well as antifeedant and larvicidal effects (Haghighian et al. 2008; Shekari et al. 2008; Hasheminia et al. 2011). Our previous study showed strong antifeedant activity of this plant extract against *G. pyloalis* larvae and significant effects on nutritional indices and biochemical compounds (Khosravi et al. 2010). The chemical constituents of the essential oil of this plant have been previously reported (Haghighian et al. 2008), but there are currently no reports on the chemical constituents of the methanolic extracts.

Plant secondary metabolites have effects through various means including acute toxicity, inhibition of enzyme activities, and discrepancy in feeding. Digestion is a phase of insect physiology of particular interest considering the economic importance of the food of insects and the fact that our most important control measures involve the action of digestive juices on poisons taken into the digestive tract. Digestive enzymes are commonly found in the salivary secretions and various regions of the digestive tract of insects. Digestive enzymes play a major role in the body of insects by converting complex food materials into smaller molecules necessary to provide energy and metabolites (Wigglesworth 1984). The metabolic enzymes are also important as they metabolize toxic compounds. The metabolic processes are hydroxylation, oxidation, deoxidation, and conjugation. Therefore, the metabolic enzymes are related to the mode of action of insecticides and the immunity of insects (Jakoby and Habig 1980).

The use of plant extracts for controlling insect pests dates back to several years ago, and thus appears a suitable area for further research. Many studies have been carried out on the potential of plant products in controlling various insect pests, and their effects on stored product pests have been studied. SenthilNathan et al. (2005) demonstrated antifeedant, larvicidal, growth regulative, ovicidal, and repellency effects of plant products on oviposition. However, the mode of action of allelochemicals in herbivorous insects is quite complex due to various active sites, and because of the difficulties in experimental design (Houseman et al. 1992). The present study focuses on the insecticidal activity of methanol leaf extract of *A. annua* against the larvae of lesser mulberry pyralid, and also effects of LC_50_ and LC_20_ doses on toxicity, larval, pupal and adult duration, mean daily fecundity, percent hatchability, and different digestive and detoxifying enzymes.

## Materials and Methods

### Mass rearing of insects

The fifth instar larvae of *G. pyloalis* were collected from mulberry orchards in the vicinity of Rasht, northern Iran. They were reared in transparent plastic jars 18 × 15 × 7 cm, and each lid had a hole covered with muslin cloth for aeration in a controlled condition (24 ± 1 °C, 75 ± 5% RH, and 16:8 L:D photoperiod). The larvae were provided with fresh mulberry leaves (Kenmochi variety) until pupation. On adult emergence, individuals were transferred to 18 × 7 cm transparent jars and provided with leaves for egg laying and cotton wool soaked in 10% honey for feeding.

### Extract preparation

The method of Warthen et al. (1984) was used. *Artemisia annua* was collected from fields, the leaves were separated and washed with distilled water and shade—dried, and finally transferred to oven (45 °C). Leaves were powdered with the help of a hand mortar. 240 grams of powdered leaf and 2400 cc methanol (Merck, www.merck.com) were placed in a glass jar and were shaken in an electric shaker for one hour and then kept under 4 °C in a refrigerator for 48 hours. They were shaken again for an additional one hour, and the solution was twice passed through Whatman No. 4 filter paper (www.whatman.com). The obtained solution was then concentrated in a rotary evaporator (Rotavapor RE 120 , Buchi Labortechnik AG, www.buchi.com) at 45 °C. The dried extract was weighed in an electric balance, and methanol was added to make a 20% w/v stock solution, which was used for making various concentrations incorporated in the experiments.

### Bioassays

**Acute toxicity of *A. annua* extract.** The bioassay experiments were performed topically on newly ecdysed fourth instar larvae of *G. pyloalis.* Prior to the experiments, larvae were starved for two hours. Initially, preparatory tests were performed to find the effective dose ranges. Four concentrations (0.21, 0.27, 0.32, 0.38 gram leaf equivalent (gle)/ mL) of toxicity. Forty larvae per concentration were used for all the experiments, and each experiment was replicated four times. 2 µL of desired concentration were topically applied on the metathoracic tergum. The controls received 2 µL of methanol in the same way. The treated larvae were allowed to eat the fresh leaves. After 24 hours the numbers of dead larvae were recorded. The LC_50_ and LC_20_ values and 95% confidence limit were calculated from probit regressions using the Polo-PC program.

The newly ecdysed fourth instar *G. pyloalis* larvae (less than 12 hours from the last molting) that were starved for two hours prior to experiments were used. 2 µL of LC_50_ and LC_20_ concentrations were topically applied on the metathoracic tergum. Fifty larvae per concentration were used for all experiments, and each experiment was replicated five times. After 24 hours the 15 surviving larvae were transferred individually to 10 × 7 × 5 cm plastic jars and were provided with fresh mulberry leaves. The jars were checked daily, the development and possible mortality was recorded, and fresh food was provided until pupation. The duration of larvae, prepupae, pupae, and adult stages were recorded in both treatment and control groups. Upon adult emergence a pair was transferred to an 18 × 7 cm jar for copulation and egg laying, where a leaf and cotton wool soaked in 10% honey solution were provided. The adult longevity of both males and females was recorded. Thirteen pairs were considered for every treatment, and the eggs laid on leaves were counted daily. The leaves and jars were changed daily. Experiments continued until the death of all adults. Eggs were observed under a stereomicroscope (Olympus SZX12, www.olympus.com) to determine the number of eggs hatched. The eggs after hatching remained very thin and transparent on the leaf surface.

### Preparation of sample for enzymatic assay

For this purpose, the fifth instar *G. pyloalis* larvae that were fed with treated food of 0.107, 0.053, 0.026 and 0.013 gle/mL methanol extract. The larvae were starved for four hours prior to the experiment. Mulberry leaf discs (radius = 8 cm) were impregnated with the above concentrations of plant extract for 10 sec and were dried at room temperature. Control leaf discs received methanol alone. The experiments were replicated four times with 10 fifth instar larvae in each replication. After 48 hours the midguts of treated and control larvae were removed by dissection under a stereomicroscope in ice cold saline buffer (0.15 M NaCl). For each sample, 10 midguts were pooled, rinsed in ice cold saline buffer, placed in a pre-cooled homogenizer and ground in 1 mL of universal buffer and then centrifuged at 10,000 g for 10 min at 4 °C. The supernatants were stored in a freezer (-20 °C) until measuring the activity of digestive enzymes. The whole body of larvae and the midguts were used for measuring the activity of glutathion stransferase (GST) and esterase enzymes respectively.

### Assay of α-amylase activity

The α-amylase activity was assayed by dinitrosalicyclic acid procedure (Bernfeld 1955) using 1% soluble starch (Merck) as substrate. 10 µL of the supernatant was incubated for 30 min at 35 °C with 40 µL universal buffer and 50 µL soluble starches. The reaction was then stopped by addition of 100 µL dinitrosalicylic acid and was kept 10 min in boiling water. Dinitrosalicyclic acid is a color reagent, and the reducing groups released from starch by α-amylase action are measured by the reduction of 3, 5-dinitrosalicylic acid. The boiling water was used for stopping the activity of α-amylase and catalyzing the reaction between dinitrosalicyclic acid and reducing groups of starch.

Absorbance was read at 540 nm after cooling in ice for five min. One unit of α-amylase activity was defined as the amount of enzyme required to produce 1 mg maltose in 30 min at 35 °C. All assays were performed in four replicates.

### Assay of protease activity

The activity of protease was determined as described by Garcia-Carreno and Haard (1993) with slight modification. 1% azocasein was used as a substrate for protease activity. Then, 10 µL of supernatant and 15 µL of buffer with 50 µL of substrate were reacted for 3 hours at 37 °C in an oven. Proteolysis was stopped by the addition of 150 µL of 10% trichloroacetic acid. Appropriate blanks, in which trichloroacetic acid was added first to the substrate, were prepared for each assay. The solutions were transferred to a refrigerator at 4 °C for 30 min, and then were centrifuged at 13,000 g for 10 min. Then 100 µL of supernatant was mixed with 100 µL NaOH and transferred to Eliza plates. The absorbance was read at 440 nm.

### Assay of lipase activity

The activity of lipase was determined by the method of Tsujita et al. (1989). 10 µL of midgut tissue extracts was mixed with 18 µL *p*-nitrophenyl butyrate (50 mM) as substrate, and 172 µL of universal buffer solution (1 M) (pH = 7) and incubated at 37 °C. The absorbance was read at 405 nm. One unit of enzyme releases 1.0 nmol of *p*-nitrophenyl per minute at pH 7.2 at 37 °C using *p*-nitrophenyl butyrate as the substrate.

### Assay of general esterase activity

The activities of general esterases were determined according to methods of Van Asperen (1962). For this purpose, αnaphtlyacetate (α-NA) and β-naphtylacetate (β-NA) (10 mM) were used as substrates. Initially, one gut was homogenized with 1000 µL 0.1 mol phosphate (pH = 7) continary Triton x-100 on the ratio of 0.01%, then the homogenized solution was centrifuged at 10,000 g for 10 min at 4 °C. The supernatant was transferred to new microtube and diluted with phosphate buffer. This solution reacts with substrate and Fast Blue RR salt (1 mM). Absorbance was recorded at 630 nm.

### Assay of glutation S-transferase activity

For determining activity of GST, the method of Habing et al. (1974) was used. In this study, 1-chloro-2, 4-dinitrobenzene (CDNB) (20 mM) was used as the substrate. First, larvae were homogenized in 20 µL distilled water, then the homogenized solution was centrifuged at 12,500 g for 10 min at 4 °C. 15 µL of supernatant was mixed with 135 µL of phosphate buffer (pH = 7), 50 µL of CDNB, and 100 µL of GST. The absorbance at 340 nm was read at intervals of nine sec in one min.

### Determination of protein

The method of Bradford (1976) was used for determining total protein, using bovine serum albumin as the standard.

### Statistical analysis

LC_50_ and LC_20_ of the toxicity bioassay were determined with Polo-PC software (LeOra Software 1987). Data from sublethal development effects and enzymes activity were compared by one—way analysis of variance (ANOVA). Difference between the treatments was determined by Tukey's multiple range tests (SAS 1997). Differences among means were considered significant at *p* ≤ 0.05.

## Results

### Bioassays

**Figure 1.**
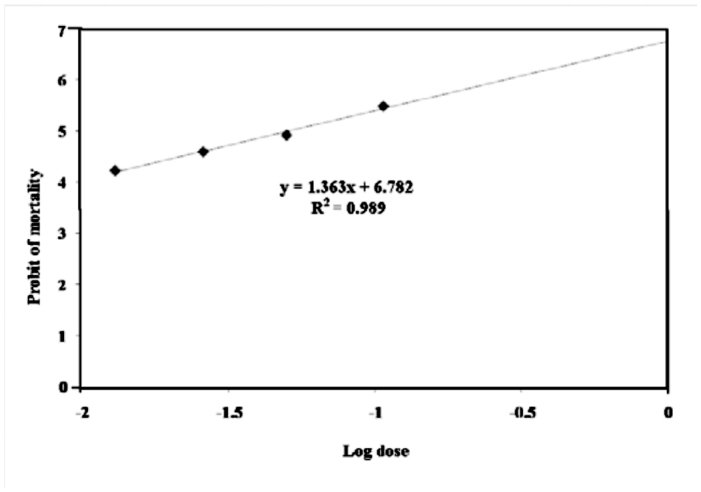
Mortality probit of *Artemisia anuua* on fourth instar *Glyphodes pyloalis* larvae. High quality figures are available online.

**Acute toxicity of *A. annua* extract.** The LC_50_ value, its confidence limits and regression slope line 24 hours after the start of the experiment were estimated. The LC_50_ of methanol extract of *A. annua* was 0.33 gle/ mL for fourth instar *G. pyloalis* larvae with confidence distance 0.29 – 0.37 (Table 1). Figure 1 shows mortality probit of *Artemisia anuua* on fourth instar *G. pyloalis* larvae.

The results of the effects of sublethal doses of *A. annua* extract on larval duration of *G. pyloalis* are depicted in Table 2. The larval duration of fourth instar larvae was increased compared with the control group (*F* = 10.02, df = 2, df = 42, *p* < 0.01). The duration of fifth instar larvae in LC_50_ treatment was increased compared with the control. However, in those larvae treated with LC_20_ dose, the duration of fifth instar larva was decreased (*F* = 33.89, df = 2, df = 42, *p* < 0.01). The duration of the prepupa stage significantly decreased in the LC_20_ treatment compared with the control. The pupal duration increased in all treatments. (*F* = 4.32, df = 2, df = 42, *p* < 0.05). The adult longevity was severely decreased in all treatments compared with the controls. The adult longevity was dose—dependent and showed a decrease with increasing concentration.

**Table 1.**
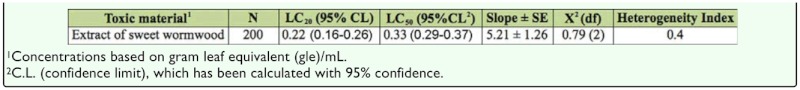
The LC_20_ and LC_50_ values, confidence limit (95%), and regression slope of methanolic extract of *Artemisia anuua* against fourth—instar larvae of *Glyphodes pyloalis* after 24 hours.

**Table 2.**
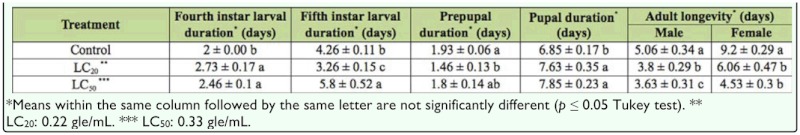
Duration of the development and longevity (mean ± SE) of *Glyphodes pyloalis* after treatment with *Artemisia anuua.*

**Table 3.**
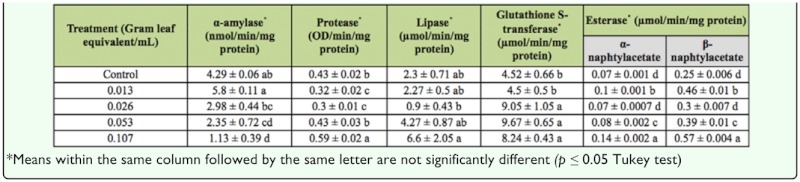
Effects *of Artemisia anuua* extract on digestive and detoxifying enzymes of *Glyphodes pyloalis* 48 hours after treatment (mean ± SE).

An extra sixth instar was observed in larvae treated with LC_50_. Total larvae entering sixth instar were 23.3 percent. All sixth instar larvae exhibited limited feeding activity and died after a few days.

The mean eggs laid (*F* = 40.74, df = 2, df = 36, *p* < 0.01) and percent hatchability (*F* = 69.19, df = 3, df = 36, *p* < 0.01) in female adults after larval treatment with sublethal doses of *A. annua* was lower compared with the control (Table 4).

**Table 4.**
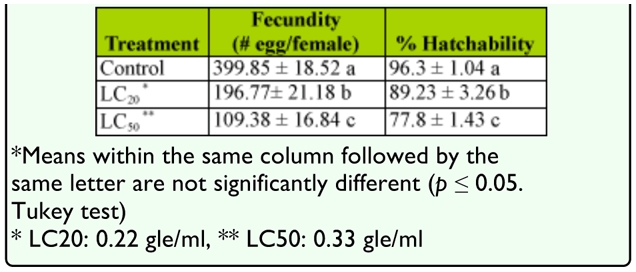
Effect of extract of Artemisia annua on fecundity and hatchability of *Glyphodes pyloalis* (mean±S.E.)

### The effect of *A. annua* extract on digestive and detoxifying enzymes

Results showed that methanolic extract of *A. annua* affected digestive enzymes of *G. pyloalis* by using oral ingestion treatment (Table 3). The activity of α-amylase in larvae treated with 0.053 and 0.107 gle/mL concentration significantly decreased. The 0.107 gle/ mL concentration depicted maximal difference with control 48 hours post—treatment (*F* = 18.19, df = 4, df = 15, *p* < 0.01). The activity of protease in fifth instar larvae treated with *A. annua* extract in 0.013 and 0.026 gle/mL decreased compared with the controls (*F* = 6.40, df = 4, df = 20, *p* < 0.01). Lipase activity increased in treated larvae and the least activity was observed in larvae treated by 0.026 gle/mL concentration (*F* = 4.14, df = 4, df = 10, *p* < 0.05).

The activity of esterase and glutathione S-transferase 48 hours post—treatment increased significantly for both substrates of enzymes. Their activities were dose—dependent, increased with increasing extract concentration, and were significantly different from the control.

## Discussion

The findings of the present investigation indicate larvicidal activity in the methanolic extract of *A. annua* against lesser mulberry pyralid. Shekari et al. (2008) reported that the larvicidal activity of methanolic extract of *A. annua* with LC_50_ values against the larvae of *Xanthogaleruca luteola* was 48%. Comparing these values with that obtained in this study reveals that the methanolic extract of *A. annua* has an excellent larvicidal effect against *G. pyloalis.*

The methanol extract of *A. annua* caused discrepancy in development of treated fourth instar larvae under sublethal doses. The larval duration was higher in fourth instar larvae compared with the control in all tested concentrations. The fifth instar larval and pupal duration was also increased in LC_50_ treatment. Many workers have reported such discrepancies in development after treatment with various plant extracts. For example, Sadek (2003) showed that pupal duration in *S. littoralis* increased under the effect of *Adhatoda vasica* extracts. Jeyabalan et al. (2003) reported that the extract of *Pelargonium citrosa* delayed larval and total stage duration of *Anophels stephensi.* Zhong et al. (2001) showed that the extracts of *Rhododedndron molle* promoted growth duration in *Pieris rapae.* Jbilou and Sayah (2008) reported delayed larval growth by *Peganum harmala* extract in *Tribolium castaneum.* In all treatments, the fourth larval and pupal duration was increased and production of F_1_ was totally prevented. Mordue et al. (2005) stated that effects of plant extract on insect growth may be due to the effects on the endocrine system and neurosecretory cells that control growth in insects.

An interesting phenomenon was the extra larval instar observed after treatment with LC_50_ dose in *G. pyloalis*, a case that could be related to hormone—like activity of the plant extract, consistent with the report of Wheeler et al. (2001).

The LC_50_ and LC_20_ dose of *A. annua* reduced fecundity and fertility of *G. pyloalis.* Essential oils of neem *Azadirakhta indica* similarly reduced fecundity of *Choristoneura rosaceana* (Lowery et al. 1996). In the same way, insects facing the sublethal doses of synthetic insecticides may also show various effects on growth, duration of life stages, pupal weight, and fecundity (Ansari et al. 2000; Huang et al. 2000).

The extract of *A. annua* caused reduction in α-amylase activity in the digestive tract of *G. pyloalis*, and this reduction was dose—dependent. Shekari et al. (2008) also reported reduction in α-amylase activity 24 hours after the treatment with methanol extracts of *A. annua* on *X. luteola*; however, 48 hours after the treatment the activity was resumed. The sublethal doses of pyrothroids also reduced α-amylase activity in *T. castaneum* (Saleem and Shakori 1987). Lee et al. (1994) also reported that some insect growth regulators reduced α-amylase activity in *Hyphantria cunea* and *Chilo supressalis.*

Proteases are groups of enzymes that hydrolyze peptide bonds. These enzymes are important in digesting food and converting protein to amino acids needed for the body. In the present study it was shown that protease activity was reduced using 0.013 and 0.026 gle/ mL concentrations of methanol extract compared with the controls 48 hours post—treatment, but increased with use of 0.107 gle/ mL concentration. Perhaps in high concentrations the activity of protease increased to degrade secondary metabolites. Low protease activity in the midgut of insects feeding on azadirachtin treated food has been reported. For example, Timmins and Reynolds (1992) concluded that azadirachtin reduces trypsin in the midgut of *Spodoptera litura* while the limonoids salanin and nimbinen had no effects (Koul et al. 1996). Zhang and Chiu (1992) also reported low protease activity in *P. rapae* larvae feeding on toosendanin treated food. Low protease, amylase, and invertase have been reported in *Preplaneta americana* by azadirachtin treated food (Paranagama et al. 2001). Researchers concluded that by way of affecting neurosecretory cells, azadirachtin caused reduction in enzyme activity. Azadirachtin prevents trypsin production in the midgut of *Manduca sexta,* and significantly affects digestive enzymes like protease, amylase and invertase in *S. litura* (Koul et al. 1996). Although it was expected that the amount of digestive enzymes should have been affected by plant extract, these reports found an increase in these enzymes. One possible explanation for this effect is that secretion of enzymes is under the control of ecdysteroidal hormones, and the plant extract had a hormonal effect (an extra larval instar) that could have affected the activity of ecdysteroids and hence the enzymes concerned. Carbohydrases have been shown to be under the control of ecdysone (Suzuki et al. 2011) and this may also be true for protease activity, which needs to be investigated.

Lipases play a major role in storage and lipid mobilization. These enzymes are also the basic components in many physiological processes like reproduction, growth, and defense against pathogens. In our study, lipase activity was not significantly different compared with the control. Senthil-Nathan (2006) showed that the azadirachtin and larvae of *Cnaphalocrosis medinalis* treated with neem had low lipase activity in the midgut. Zibaee and Bandani (2009) also reported low lipase activity in *Eurygaster integriceps* treated by *A. annua* extract.

In the present study, the activity of glutathione S-transferase in the larvae treated with *A. annua* extract was increased compared with the control. Zibaee and Bandani (2010) reported that activity level of GST in *E. integriceps* treated with *A. annua* increased. This shows that glutathione S-transferase plays a role in the detoxification or in the metabolism of *A. annua* extract. In general, the conjugated products are ionic compounds with more polarity that are easily defecated, and are usually less toxic than the primary compounds. Although conjugated compounds cause lower toxicity, sometimes the conjugated complex breaks and releases toxic components. The alkaloids of *Sophora alopecuroides* prevented glutathione S-transferase activity in the diamond back moth (Luo and Zhang 2003). In the present study, it was shown that esterase enzyme activity in the fifth instar larvae of *G. pyloalis* was increased 48 hours post—treatment. Liu et al. (2008) reported that GST and general esterase activity didn't change in the larvae of *O. furnacalis* after feeding on fraxinellone—treated food. Boivin et al. (2001) reported that in *Cydia pomonella* high levels of energy consumption occurs during detoxification that leads to lower or higher larval duration or reduction of reproductive performance (Boivin et al. 2001). However, the results of our study showed that *A. anuua* extract does not have an inhibitory effect on detoxification enzymes of *G. pyloalis* after the consumption of *A. anuua* extract. This suggests that esterase and GST are involved in detoxification or metabolism of the compound.

## Conclusion

Biopesticides with plant origins have been given new importance in recent years for their use against several insect species. This work shows that methanol extract of *A. annua* possesses potent toxic and developmental inhibiting effects in *G. pyloalis.* Moreover, compounds present in the extract of *A. annua* affect the activity of digestive and detoxification enzymes. Physiological analysis would be particularly informative to gain insight into the efficiency of a safe management process.

## **Abreviations**

GST,: glutathion S-transferase;
**gle,**: gram leaf equivalent
